# Geographic variation in health insurance benefits in Qianjiang District, China: a cross-sectional study

**DOI:** 10.1186/s12939-018-0730-3

**Published:** 2018-02-05

**Authors:** Yue Wu, Liang Zhang, Xuejiao Liu, Ting Ye, Yongfei Wang

**Affiliations:** School of Medicine and Health Management, Tongji Medical College, Huazhong University of Science and Technology, 13 Hangkong Road, Wuhan, China

**Keywords:** Health insurance, Universal health coverage, Geographic variation, *Theil index*, Geographic information system, Cross-sectional study

## Abstract

**Background:**

Health insurance contributes to reducing the economic burden of disease and improving access to healthcare. In 2016, the Chinese government announced the integration of the New Cooperative Medical Scheme (NCMS) and Urban Resident Basic Medical Insurance (URBMI) to reduce system segmentation. Nevertheless, it was unclear whether there would be any geographic variation in health insurance benefits if the two types of insurance were integrated. The aim of this study was to identify the potential geographic variation in health insurance benefits and the related contributing factors.

**Methods:**

This cross-sectional study was carried out in Qianjiang District, where the NCMS and URBMI were integrated into Urban and Rural Resident Basic Medical Insurance Scheme (URRBMI) in 2010. All beneficiaries under the URRBMI were hospitalized at least once in 2013, totaling 445,254 persons and 65,877 person-times, were included in this study. Town-level data on health insurance benefits, healthcare utilization, and socioeconomic and geographical characteristics were collected through health insurance system, self-report questionnaires, and the 2014 Statistical Yearbook of Qianjiang District. A simplified *Theil index* at town level was calculated to measure geographic variation in health insurance benefits. Colored maps were created to visualize the variation in geographic distribution of benefits. The effects of healthcare utilization and socioeconomic and geographical characteristics on geographic variation in health insurance benefits were estimated with a multiple linear regression analysis.

**Results:**

Different *Theil index* values were calculated for different towns, and the *Theil index values* for compensation by person-times and amount were 2.5028 and 1.8394 in primary healthcare institutions and 1.1466 and 0.9204 in secondary healthcare institutions. Healthcare-seeking behavior and economic factors were positively associated with health insurance benefits in compensation by person-times significantly, meanwhile, geographical accessibility and economic factors had positive effects (*p* < 0.05).

**Conclusions:**

The geographic variation in health insurance benefits widely existed in Qianjiang District and the distribution of health insurance benefits for insured inpatients in primary healthcare institutions was distinctly different from that in secondary healthcare institutions. When combining the NRCM and URMIS in China, the geographical accessibility, healthcare-seeking behavior and economic factors required significant attention.

## Background

Health insurance has emerged as the main approach to improving financial risk protection and access to quality healthcare [1–3]. In 2005, all WHO member states made a commitment to achieve universal health coverage, which is a collective expression of the belief that all people should have access to healthcare services without risk of financial ruin [4]. Numerous countries have taken measures to improve universal health coverage and have made great progress, such as Mexico [5] and Rwanda [6].

In China, health insurance has nearly reached universal coverage, covering more than 98% of individuals. However, segmentation has been severe for a long time among New Cooperative Medical Scheme (NCMS), Urban Resident Basic Medical Insurance (URBMI), and Urban Employee Basic Medical Insurance (UEBMI) which cover different population groups. These three identity-based, district-varied health schemes have led to rural-urban variation in health insurance and have affected access to healthcare [7]. Furthermore, there are also great disparities in the reimbursements [8]. Considering the serious segmentation of the health insurance system and in order to improve the equity, sustainability and efficiency of NCMS and URBMI, the decision to integrate the NCMS and URBMI was announced in China in 2016 [9].

However, it is unclear whether the integration of NCMS and URBMI has impacted geographic variation in medical services utilization. It refers to variation of medical services use of a population according to the geographically defined unit [10]. Geographic variation in medical services utilization is explained by both patient characteristics and supplier characteristics and has remained a major topic in research field. It is generally known that gaps in economic development may affect the utilization of medical services, residents with higher income and educational status may utilize more healthcare services than others [11]. And geographical accessibility is one of the possible determinants of medical services utilization [12–14]. In this study, we exploited geographic variation in health insurance benefits to reflect the geographic variation in medical services utilization.

Since John Snow first considered geographic variations in health by mapping the location of cholera cases around water pumps in mid-1800s, numerous studies have been conducted on geographic variation in at least four aspects of health. Firstly, many studies examined variation in the geographic distribution of diseases, including prevalence or incidence rate, as well as the survival and mortality rates of several diseases [15–20]. Secondly, some studies focused on health resource allocation. In 1999, Mick SS et al. studied the geographic distribution of medical graduates [21]. Thirdly, some researchers investigated geographic variation in healthcare services, including cancer screening or other services [22–25]. Fourthly, some studies examined geographic variation in healthcare expenses [26–29]. By analyzing the medication information of the elderly under Medicare Part D, Zhang Y et al. concluded that the geographic variation in prescription safety was larger than that in drug spending and that there was no association between high drug spending and medical care quality [30, 31].

To determine whether there will be any obvious geographic variation in health insurance benefits worth studying upon the integration of NCMS and URBMI. We conducted a cross-sectional study in Qianjiang District in China in 2013, where NCMS and URBMI were integrated into a basic medical insurance called the Urban and Rural Resident Basic Medical Insurance Scheme (URRBMI) in 2010, to shed light on this issue as well as to provide evidence for health insurance integration. This study aimed to identifying geographic variations in health insurance benefits and the contributing factors. We had three overall objectives in the current study: (1) to produce a descriptive analysis of variation in health insurance benefits; (2) to map the geographic distribution of variation in health insurance benefits; and (3) to analyze the potential factors influencing geographic variation in health insurance benefits.

## Methods

### Study setting and sample

**Fig. 1:**
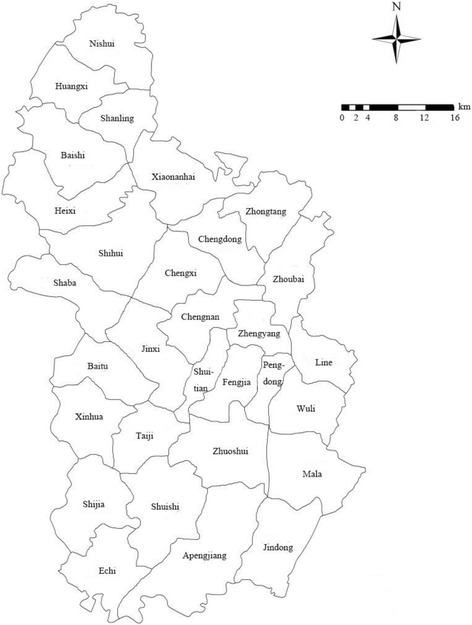
Qianjiang administrative map

A cross-sectional study was conducted in Qianjiang District in China in 2013. Our study sample comprises all beneficiaries under the URRBMI who were hospitalized at least once in 2013, totaling 445,254 persons and 65,877 admissions. In this study, primary healthcare institutions consist of all township health institutions, community health centers and other clinics. Other hospitals are classified as secondary healthcare institutions. In this district, as shown in Fig. 1, the urban areas include Chengdong, Chengxi, Chengnan, Zhengyang, Zhoubai, and Zhuoshui. The district center represents Chengdong, Chengxi, and Chengnan. There are several arterial roads, primarily north-south highways (G319, G65).

### Unit of analysis

Based on previous studies of geographic variation, administrative units have frequently been used for geographic variation studies [10, 26]. Furthermore, Verena Vogt et al., who examined geographic variation in the use of cancer screening at the district level, proposed that analysis on a smaller geographical scale could result in more reliable conclusions [32]. Therefore, we analyzed data at the town level in this study.

### Statistical analysis

#### Theil index

Concentration and spatial variation measures are usually performed by means of a variety of indices, i.e. the Gini index, the *Theil index*, the concentration index, and Moran’s I. *Theil index* is considered the simplest variation indicator for the reason that it does not depend on any additional parameter [33], hence, we calculated the *Theil index* for health insurance compensation by person-times and compensation by amounts at the town level to analyze geographic variation in health insurance benefits. Geographic variation was estimated using the following simplified formula:

$$ \boldsymbol{T}=\sum \limits_{\boldsymbol{i}=\mathbf{1}}^{\boldsymbol{n}}{\boldsymbol{P}}_{\boldsymbol{i}}\boldsymbol{\log}\frac{{\boldsymbol{P}}_{\boldsymbol{i}}}{{\boldsymbol{Y}}_{\boldsymbol{i}}} $$

Where *P*_*i*_ equals the proportion of the insured population of region *i* in the whole insured population, and *Y*_*i*_ represents the proportion of compensation by person-times or by amounts of region *i* in the whole compensation by person-times or by amounts. If *P*_*i*_ = *Y*_*i*_, then *T*_*i*_ equals 0, which indicates that there is no inequality for this region. If *P*_*i*_ > *Y*_*i*_, then *T*_*i*_ > 0, his region is not in favor of compensation; the bigger the value, the greater the disadvantage region *i* suffers. If *P*_*i*_ < *Y*_*i*_, then *T*_*i*_ < 0, this region is in favor of compensation; the smaller the value, the greater the advantage region *i* enjoys.

#### Spatial interpolation analysis

The geographic distribution of health insurance benefits based on the *Theil index* values was displayed by the colored choropleth map using the spatial interpolation analysis with the Geographic Information System [34]. With this method, the closer the points are in the space, the more likely it is that these points have similar characteristics. With the spline function, points were interpolated into the grid surface, and data of discrete points were converted into continuous surface data.

#### Multiple linear regression

In order to clarify the spatial variation of health insurance benefits, the non-spatial regression, ordinary least squares (OLS), was firstly used with the *Theil index* value as the dependent variable. Considering the study framework of Anderson’s healthcare utilization model [35], unit of analysis and data availability, we selected ability of healthcare delivery, healthcare-seeking behavior of the insured, geographical accessibility of healthcare, and economic factors to analyze the potential factors influencing geographic variation of health insurance benefits.

1. The ability of healthcare delivery was measured by healthcare staff density (the number of healthcare staff per 1000 residents), the density of actual open beds (the number of actual open beds per 1000 residents) and the availability of lower abdominal surgery (available = 1). The number of healthcare staff and actual open beds, as well as the availability of lower abdominal surgery, were investigated using questionnaires for the township health centers. Population for each town was collected through *the 2014 Statistical Yearbook of Qianjiang District*.
2. The healthcare-seeking behavior of the insured was computed as the proportion of compensation by person-times in primary healthcare institutions that accounted for the overall compensation by person-times in each town and the data were collected from the basic health insurance system.
3. The geographical accessibility of healthcare was estimated based on travel time from the town government to the Qianjiang Central Hospital, which is the best hospital in that district, by car using Google Maps [36, 37].
4. The town-level economic factor was represented by the per-capita net income at the town level in RMB (¥) in 2013 and was collected from *the 2014 Statistical Yearbook of Qianjiang District*.

The density of healthcare staff, density of actual open beds, geographical accessibility of healthcare and town-level economic factor were conducted by standardized normal Z transformation to eliminate dimension so that data had same caliber.

Moran’s I for OLS residuals were tested in order to find the necessity of taking spatial dependencies into consideration. In the current study, it demonstrated that no significant spatial autocorrelation existed (For compensation by person-times as the dependent variable: Moran’s *I* = − 0.021, *p* = 0.397 > 0.05; For compensation by amounts as the dependent variable: Moran’s *I* = − 0.049, *p* = 0.387 > 0.05) and there was no need to conduct spatial regression analysis [38, 39].

All maps were displayed using ArcGIS 10.2. *Theil index*, Moran’s I and multiple linear regression analysis were performed using Stata Version 13.0.

## Results

### Theil index value

The *Theil index* values showed that there was great geographic variation of health insurance benefits in both compensation by person-times and amount, and the geographic variation distribution was divergent for hospitalization types, presented in Table 1. The total *Theil index* values for compensation by person-times and amount were up to 0.5886 and 0.7074 respectively. However, the variation resulted from hospitalization between primary healthcare institutions and secondary healthcare institutions were much more severe, *Theil index* values for compensation by person-times and amount were 2.5028 and 1.8394 in primary healthcare institutions, and 1.1466 and 0.9204 in secondary healthcare institutions. Besides, the numbers of which townships were in favor of health insurance benefits also demonstrated the different distribution, for 18 towns were in favor of compensation by person-times for hospitalization in primary healthcare institutions while the number was only 9 for that in secondary healthcare institutions, and 16 towns were in favor of compensation by amounts for hospitalization in primary healthcare institutions while the number was only 10 for that in secondary healthcare institutions.

**Table 1:** *Theil index* values of the compensation number and amount of insured inpatients

Town	The insured	The whole district				Primary healthcare institutions				Secondary healthcare institutions			
		Number	*T*_*p*_	Amount	*T*_*c*_	Number1	*T*_*p1*_	Amount1	*T*_*c1*_	Number2	*T*_*p2*_	Amount2	*T*_*c2*_
Chengdong	24,000	3543	0.0052	6,794,882	− 0.4080	712	1.7902	689,928	0.4447	2831	−0.7887	6,103,636	−0.5267
Chengnan	19,917	2961	−0.0093	5,796,492	−0.3921	623	1.3828	419,902	0.9714	2338	−0.6451	5,377,400	−0.5532
Chengxi	19,800	3000	−0.0459	5,174,217	−0.1819	902	0.6486	586,300	0.3096	2098	−0.4435	4,588,326	−0.2549
Zhengyang	13,981	2627	−0.3259	4,422,418	−0.3889	1063	−0.2405	499,610	−0.0377	1564	−0.3872	3,922,512	−0.4407
Zhoubai	23,000	4386	−0.5693	6,596,934	−0.4202	1920	−0.6053	919,680	−0.3142	2466	−0.5417	5,679,198	−0.4385
Fengjia	22,456	3777	−0.2809	6,401,660	−0.3968	1649	−0.3102	1,035,572	−0.6191	2128	−0.2584	5,366,816	−0.3567
Xiaonanhai	8569	1377	−0.0690	1,760,066	0.1225	826	−0.3458	409,696	−0.2664	551	0.2255	1,350,501	0.2119
Line	13,122	2175	−0.1454	3,056,112	0.0268	1141	−0.3976	621,845	−0.3967	1034	0.0851	2,434,036	0.1159
Apengjiang	25,025	3779	−0.0499	5,560,764	0.1659	1880	−0.4013	825,320	0.1284	1899	0.2543	4,736,106	0.1721
Shihui	19,718	2709	0.1425	4,338,713	0.1496	1174	0.1310	709,096	−0.0653	1535	0.1513	3,630,275	0.1886
Heixi	20,202	2145	0.6538	3,840,949	0.4412	705	1.1869	331,350	1.4800	1440	0.3286	3,509,280	0.3078
Huangxi	12,505	1466	0.2839	2,370,757	0.2766	761	0.0564	522,046	−0.2234	705	0.4895	1,848,510	0.3874
Nishui	12,848	1624	0.1973	2,057,001	0.4960	904	−0.1239	327,248	0.3897	720	0.5105	1,729,440	0.5154
Jinxi	13,659	2049	−0.0184	3,326,494	−0.0316	993	−0.1753	805,323	−0.7039	1056	0.1140	2,520,672	0.1275
Mala	15,882	2565	−0.1357	3,825,077	−0.0195	1408	−0.5112	737,792	−0.4492	1157	0.2246	3,086,876	0.0680
Zhuoshui	16,377	3000	−0.3412	5,230,374	−0.4709	1205	−0.2294	718,180	−0.3712	1795	−0.4209	4,512,630	−0.4875
Shijia	13,876	1922	0.0892	3,121,151	0.0755	869	0.0238	485,771	−0.0096	1053	0.1410	2,635,659	0.0905
Echi	12,120	1410	0.2842	2,267,085	0.2839	625	0.2505	288,125	0.4492	785	0.3104	1,978,985	0.2579
Zhongtang	14,620	1822	0.2447	3,268,018	0.0885	606	0.6136	345,420	0.5506	1216	0.0178	2,922,048	0.0227
Pengdong	8178	1715	−0.2782	2,138,677	−0.0757	984	−0.5069	423,120	− 0.3173	731	−0.0475	1,715,657	−0.0259
Shaba	14,334	2188	−0.0436	3,777,906	−0.1436	733	0.3079	400,951	0.3038	1455	−0.2611	3,377,055	−0.2077
Baishi	16,911	1826	0.5196	3,435,822	0.2598	675	0.7720	497,475	0.2753	1151	0.3513	2,938,503	0.2572
Shanling	9468	1264	0.0949	1,535,344	0.3537	732	−0.1783	333,792	−0.0130	532	0.3737	1,201,788	0.4340
Taiji	12,045	1739	0.0288	2,790,462	0.0309	827	−0.0874	380,420	0.1126	912	0.1250	2,410,416	0.0173
Shuitian	10,035	1229	0.1850	2,084,185	0.1326	452	0.3398	208,824	0.5022	777	0.0823	1,875,678	0.0812
Baitu	10,962	1294	0.2415	1,863,312	0.3591	709	−0.0157	432,490	−0.1354	585	0.4878	1,430,910	0.4726
Jindong	11,152	1499	0.1044	2,390,102	0.1132	783	−0.1052	461,187	−0.1889	716	0.2951	1,928,904	0.1746
Wuli	11,029	1806	−0.1091	2,555,192	0.0282	970	−0.3464	462,690	−0.2023	836	0.1133	2,092,508	0.0732
Shuishi	10,763	1615	−0.0148	2,065,960	0.2250	864	−0.2422	343,008	0.0912	751	0.1975	1,722,794	0.2499
Xinhua	8700	1365	−0.0498	2,050,697	0.0076	684	−0.1781	255,132	0.1443	681	0.0621	1,795,116	−0.0135
total	445,254	65,877	0.5886	105,896,823	0.7074	28,379	2.5028	15,477,293	1.8394	37,498	1.1466	90,422,235	0.9204

Note: Number, Number1, Number2, the compensation number; *T*_*p*_, *T*_*p1*_, *T*_*p2*_, the *Theil index* value of the compensation by person-times. Amount, Amount1, Amount2, the compensation by amounts; *T*_*c*_, *T*_*c1*_, *T*_*c2*_, the *Theil index* value of the compensation by amounts. The insured, the number of the insured population in the Qianjiang District

### Spatially interpolated and geographic variation

**Fig. 2:**
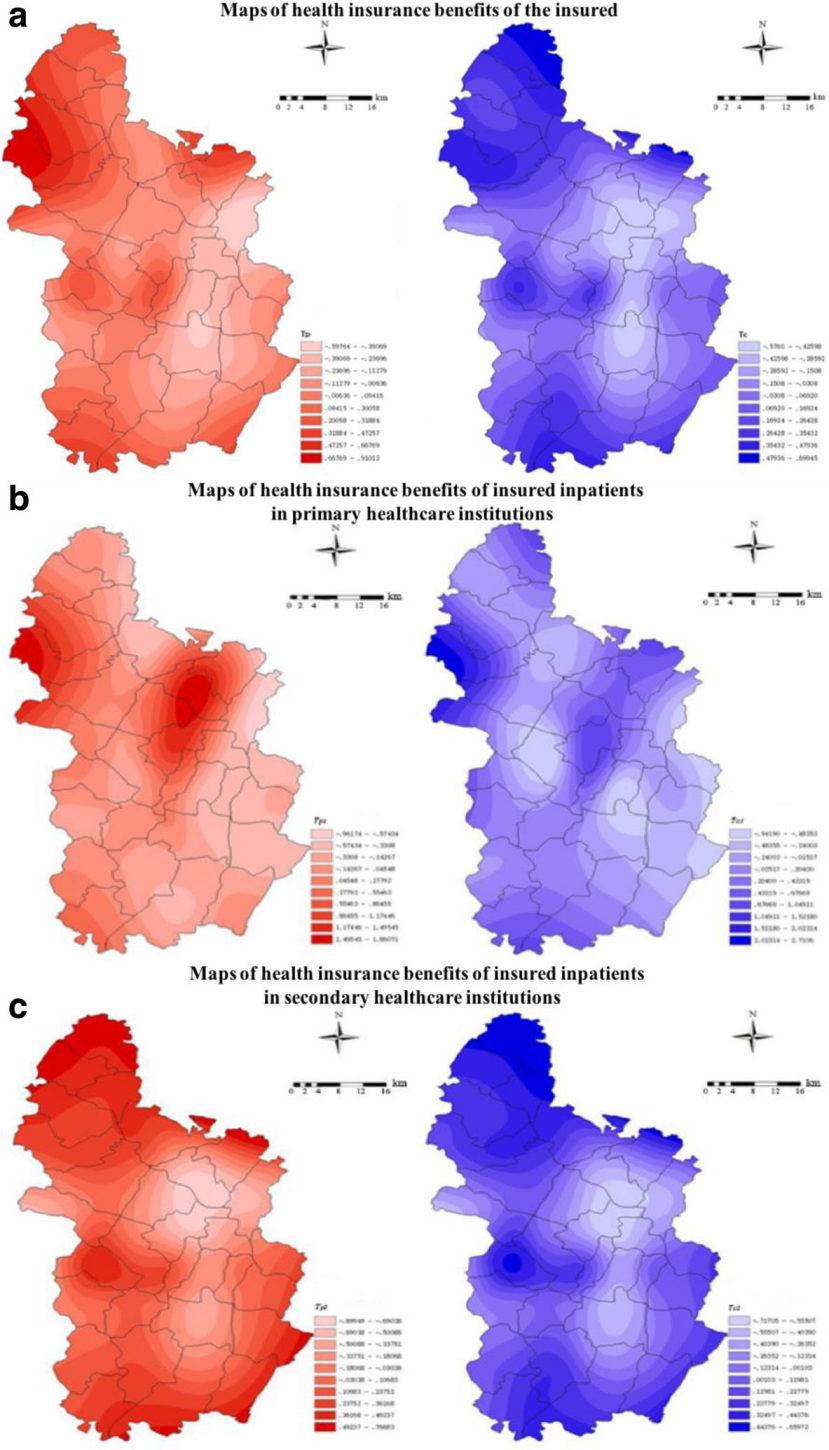
*Theil index* of health insurance benefits. Note: Panel **abc** describe the maps of health insurance benefits of the whole insured inpatients, the insured inpatients in primary healthcare institutions, and the insured inpatients in secondary healthcare institutions, respectively. The red maps in the left-side represent health insurance benefits of compensation by persontimes, the blue maps in the right-side represent health insurance benefits of compensation by amounts

The spatially interpolated surface of health insurance compensation by person-times and amount permits a more visual image of regional patterns of health insurance benefits in Qianjiang. As shown in Fig. 2, the higher the *Theil index* value, the deeper the color and the more disadvantage experienced by the region. It was obvious that the distribution of health insurance benefits for insured inpatients in primary healthcare institutions was distinctly different from that in secondary healthcare institutions.

For health insurance compensation by person-times as shown in Part A, the deepest colors appeared in remote and broader areas western and southern, including Heixi Town, and the lightest colors district center and along the north-south highways, consisting of Zhoubai, Zhengyang, Fengjia, Pengdong, and Zhuoshui. However, in addition to the similar geographic distribution, there were deep colors in the north of Nishui Town for compensation by amounts.

For primary healthcare institutions shown in Part B, the deeper colors were distributed west of Heixi and around the district center, including Zhongtang, Chengdong and Chengnan, and the lightest colors were distributed across most of this district for health insurance benefits.

By contrast, for secondary healthcare institutions as shown in Part C, there were deeper colors in the remote and border areas northwestern, western and southern for the compensation by person-times and amounts. The lightest colors presented a more concentrated distribution in the eastern areas, which were seen around the district center and along the north-south highways.

### Results of regression analysis

As shown in Table 2, the per-capita net income at the town level that measured economic factor had significant negative effect on *Theil index* values of both compensation by person-times and compensation by amounts, which demonstrated that regions considerably richer enjoyed more healthcare insurance benefits. This finding aligns with most prior studies [11, 40].

**Table 2:** Regression coefficients and standard errors

Variables	T_*p*_ coefficients (SE)	T_*c*_ coefficients (SE)
ZStaff	−0.037 (0.040)	−0.027 (0.048)
ZBed	0.006 (0.034)	−0.016 (0.041)
Availability	0.054 (0.078)	0.013 (0.093)
SeekBeha	−0.088*** (0.015)	−0.025 (0.017)
ZAccessibility	0.063 (0.042)	0.122** (0.050)
ZEconomic	−0.092** (0.04)	−0.110** (0.049)
Constant	0.593*** (0.101)	0.186 (0.121)

Note: **p < 0.05, ***p < 0.001; *SE* standard error, *ZStaff* the standardized density of healthcare staff, *ZBed* the standardized density of actual open beds, *Availability* the availability of lower abdominal surgery, *SeekBeha* the healthcare-seeking behavior of the insured, *ZAccessibility* the standardized geographical accessibility of healthcare, *ZEconomic* The town-level economic factor

Patients’ healthcare-seeking behavior represented by the proportion of hospitalization admissions in primary health centers including township health centers and community health center, was positively related to benefits in compensation by person-times, while, this kind of relationship could not be testified in compensation by amounts. On the contrary, the significantly negative coefficient for the standardized geographical accessibility of healthcare in the OLS model demonstrated that the central area where most of the secondary healthcare institutions and both political and economic center located had an advantage in health insurance compensation by amounts, while, no such relationship could be found in compensation by person-times. It testified that the central region consumed more health insurance resources than the remote areas did, which actually aligns with our observation that the residents in central part, were more likely to hospitalize in secondary hospital where the expense were much higher than the average.

The findings showed that the ability of healthcare delivery represented by the density of healthcare staff, actual open beds and the availability of lower abdominal surgery was an inexplicable explanatory variable for health insurance benefits both in compensation by person-times and amount.

The two OLS models had comparatively high goodness of fit [41]. R^2^ were 0.742 and 0.656 for compensation by person-times and amount respectively. The regression residuals for each model obeyed the normal distribution (for Tp: *p* = 0.200 > 0.05; for Tc: *p* = 0.201 > 0.05). Collinearity diagnosis results showed that the Variance inflation coefficient (VIF) for each independent variable was smaller than 10, which demonstrated the little possibility of collinearity.

## Discussion

In this study, we found that towns had different *Theil index* values of health insurance benefits and that there was great geographic variation in health insurance benefits. Furthermore, economic factor had a significant positive influence on health insurance benefits in Qianjiang District.

Our results clearly revealed the characteristics of geographic variation in health insurance benefits. There was smaller geographic variation in compensation by person-times than in compensation by amounts for all the insured inpatients. However, for primary healthcare institutions, the geographic distribution of variation in compensation by person-times and amounts were almost the same: insurance benefits were sparse in the western areas and district centers for primary healthcare institutions. In contrast, for secondary healthcare institutions, insurance benefits were concentrated in the district center and the regions along the north-south highways, which was nearly the same as the geographic distribution of the variation in health insurance benefits in the whole district. The distribution of geographic variation proved the necessity of exploring the factors from the perspective of patient characteristics and supplier characteristics.

Our results demonstrated that economic factor was positively associated with the health insurance benefits significantly. The distribution of income within society is one of the main factors affecting health and its services [42, 43], and those in the high-income group may be more likely to follow doctors’ recommendations, seek and use inpatient services, and may benefit more from central subsidies than the low-income group under the same basic health insurance [44]. Generally, the residents in and around the district center have higher incomes. Therefore, residents located in the district center may have more healthcare service utilization and benefit more from the same basic health insurance than others.

However, the healthcare-seeking behavior of the insured was only negatively associated with the *Theil index* values of health insurance compensation by person-times. This may be in accordance with the focus on primary care delivery; the “tiered health service delivery” and the reimbursement strategy of basic health insurance are inclined toward primary healthcare institutions in the rural healthcare system in China. Nevertheless, the percentage was not associated with the *Theil index* value of compensation by amounts.

Our results demonstrated that geographical accessibility of Qianjiang Central Hospital medical services led to geographic variation in health insurance benefits in the perspective of compensation by amounts. Actually, it is consistent with our observation that residents in and around the central part of this district and near the north-south highways may experience more convenient traffic, which leads to less travel time and considerably better healthcare services provided by the secondary healthcare institutions, hence resulting in more utilization of healthcare services of high expenses. The association between both the road distribution and traffic convenience and the geographical accessibility of social resources has already been demonstrated [45], so has the relationship between geographical accessibility and healthcare-seeking behavior and services utilization [46, 47]. However, the geographic accessibility has insignificant effect on health insurance benefits in compensation by person-times, for the reason that the geographic accessibility in this study was represented by the shorted time to arrive in Qianjiang Central Hospital instead of the nearest healthcare service, which is distinctly different from the definition in prior studies [46, 47].

However, we found no statistical association between three indicators of healthcare delivery and geographic variation in health insurance benefits. This may have been because we selected only three typical indicators to represent healthcare delivery at the town level, and we did not have enough evidence to guarantee that these indicators could measure healthcare service delivery appropriately, accurately, and effectively in this district.

This study has several potential limitations. First, individual and family information of the insured inpatients was ignored, for their demographic characteristics, socio-economic characteristics, diseases type or disease severity were not taken into consideration. Second, geographic variation in health insurance benefits was analyzed at the township level in this study. Heterogeneity of towns were ignored as township is the smallest administrative unit in China containing its own area, geography and population. Zip code [48] and healthcare service unit [49, 50] need to be investigated in our further study as they were selected in some previous studies. Finally, no pre-after analysis was conducted in the current study which led to the unavailability of demonstrating the effect of integration of the two kinds of health insurance on geographic variation of health insurance benefits.

Despite the above limitations, our findings are of great importance to health insurance policymaking. The concept that: “coverage should be for everyone”, one of the features of universal health coverage, is built on the foundation that good healthcare services be available from the health resources located close to people, regardless of income level, without unaffordable out-of-pocket payments [51]. When combining the NRCM and URMIS, we need to attach great importance to the system-wide or district-wide geographic variation in health insurance benefits and the contributing factors, namely, the geographic accessibility of healthcare and economic factors.

## Conclusion

This study was the first to examine geographic variation in health insurance benefits in China. In this study, the *Theil index* values were differed by town, and there was great geographic variation at the town level. Moreover, the geographic accessibility of healthcare, healthcare-seeking behavior and economic factors may be of great significance. The next step in our further research may be to expand the sample areas under the pilot of the integration of the NCMS and URBMI.

## Abbreviations

Availability: the availability of lower abdominal surgery
NCMS: New Cooperative Medical Scheme
OLS: Ordinary Least Squares
SeekBeha: the healthcare-seeking behavior of the insured
UEBMI: Urban Employee Basic Medical Insurance
URBMI: Urban Resident Basic Medical Insurance
URRBMI: Urban and Rural Resident Basic Medical Insurance Scheme
ZAccessibility: the standardized geographical accessibility of healthcare
ZBed: the standardized density of actual open beds
ZEconomic: The town-level economic factor
ZStaff: the standardized density of healthcare staff
